# Prospects for control of emerging infectious diseases with plasmid DNA vaccines

**DOI:** 10.1186/1476-8518-7-3

**Published:** 2009-09-07

**Authors:** Ronald B Moss

**Affiliations:** 1 Vical Inc. San Diego, CA, USA; 2 NexBio, Inc, San Diego CA, USA

## Abstract

Experiments almost 20 years ago demonstrated that injections of a sequence of DNA encoding part of a pathogen could stimulate immunity. It was soon realized that "DNA vaccination" had numerous potential advantages over conventional vaccine approaches including inherent safety and a more rapid production time. These and other attributes make DNA vaccines ideal for development against emerging pathogens. Recent advances in optimizing various aspects of DNA vaccination have accelerated this approach from concept to reality in contemporary human trials. Although not yet licensed for human use, several DNA vaccines have now been approved for animal health indications. The rapid manufacturing capabilities of DNA vaccines may be particularly important for emerging infectious diseases including the current novel H1N1 Influenza A pandemic, where pre-existing immunity is limited. Because of recent advances in DNA vaccination, this approach has the potential to be a powerful new weapon in protecting against emerging and potentially pandemic human pathogens.

## 

Throughout recorded history, infectious diseases have plagued human existence. One effective approach to limiting these diseases has been vaccination. For example, in a recent report by Roush and colleagues at the U.S. Centers for Disease Control and Prevention (CDC), ever since the introduction of vaccines the incidence of infectious diseases like diphtheria, mumps, pertussis, tetanus, hepatitis A and B, *Haemophilus *influenza and varicella zoster has declined by more than 80% in the U.S [1]. Furthermore, after the introduction of vaccines, large scale transmission of measles, rubella, and polio has been eliminated in the U.S., while smallpox has been eradicated worldwide. However, new emerging infectious pathogens such as HIV (human immunodeficiency virus), SARS coronavirus (severe acute respiratory syndrome virus), and highly pathogenic avian influenza (H5N1) viruses have adapted strategies to rapidly change their genetic compositions. As the influenza pandemic of 1918 (H1N1) killed approximately 20 to 50 million people worldwide, massive disease and death is similarly feared from newly emerging pathogens. In addition, the current novel swine derived H1N1 pandemic further exemplifies the need for a rapid and effective vaccine against emerging pathogens [2]. Thus a vaccination strategy to control emerging diseases will require a more effective and rapid response than available from conventional approaches such as live-attenuated vaccines, inactivated vaccines, or protein subunit vaccines. Plasmid DNA vaccines, as reviewed in this article, may be an option to effectively combat current emerging infectious diseases.

## History of DNA Vaccines

Almost 20 years ago, Malone and Felgner at Vical Incorporated, and Wolff and colleagues at the University of Wisconsin, demonstrated that mRNA and closed loops of double-stranded DNA (plasmids) injected into muscle tissue could be taken up by cells at the administration site (transfection) resulting in the production (expression) of proteins not normally made by the host cell [3]. It was soon realized that this approach could be utilized for both gene therapy as well as vaccine applications, and thus the field of DNA vaccines was born.

Shortly after these original observations, many groups including those led by Liu and colleagues at Merck Research Laboratories [4], Weiner and colleagues at University of Pennsylvania [5], Johnston and colleagues at University of Texas [6], Robinson and colleagues at University of Massachusetts [7], and Hoffman and colleagues at Naval Medical Research Center [8], demonstrated that immunization with DNA could result in the production of foreign proteins or antigens that stimulate the immune system resulting in protection from or amelioration of infectious diseases in animal models. Development in this area has greatly advanced over the years and human clinical trials of DNA vaccines have now been conducted against various infectious pathogens including the malaria parasite, dengue viruses, cytomegalovirus (CMV), Ebola virus, seasonal influenza viruses, avian or pandemic influenza viruses, West Nile virus (WMV), SARS coronavirus, hepatitis B virus, and HIV.

### Sidebar 1: Mechanism of plasmid DNA vaccines

**Figure 1:**
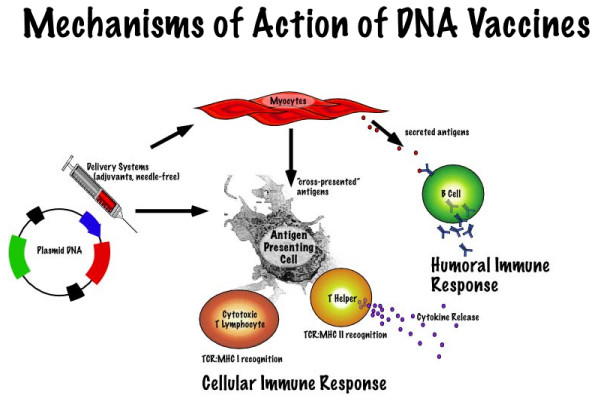
**Proposed mechanism of DNA vaccines**.

The precise mechanism of the induction of immunity after pDNA vaccination is complex [9] and multi-factorial (Figure 1).

It is thought that after immunization, transfected muscle cells may produce antigen or foreign proteins that then directly stimulate B cells of the immune system, which in turn produce antibodies. Transfected muscle cells could possibly transfer the antigen to so-called antigen presenting cells (as demonstrated by cross priming) which then transport the proteins via distinct pathways (the MHC I for CD8+T cells or MHC II for CD4+T cells) that result in the display of different processed fragments of expressed proteins (antigens). Finally, direct transfection of antigen presenting cells (such as dendritic cells) with subsequent processing and display of MHC-antigen complexes may also occur. Because the process of antigen production by host cells after DNA vaccination mimics the production of antigens during a natural infection, the resulting immune response is thought to be similar to the type induced by pathogens. Indeed, DNA vaccination generates antigens in their native form and with similar structure and function to antigens generated after natural infection.

### Sidebar 2: Full Circle - Plasmid DNA Design

**Figure 2:**
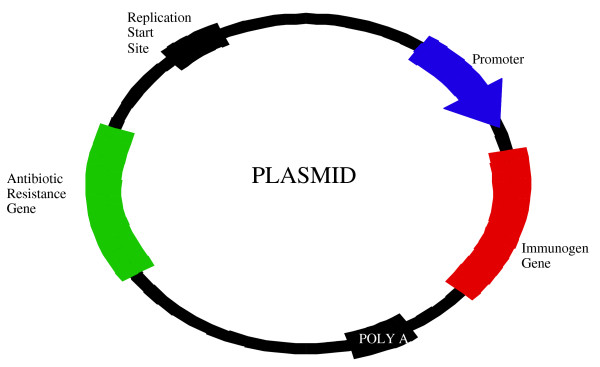
**Components of DNA Vaccines**.

A plasmid used in DNA vaccination (Figure 2) contains a gene encoding an antigen of the target pathogen (immunogen gene). Expression of the protein antigen is "turned on" in the host cell by a promoter, and "turned off" by a terminator (a polyadenylation signal sequence, generally referred to aspoly-A). Other genes such as the bacterial origin of replication sequence and an antibiotic resistance gene are incorporated for manufacturing purposes. The resulting plasmid is a stable, self-contained unit that can be manufactured by consistent and scalable bacterial fermentation and purification processes.

### Sidebar 3: Manufacturing DNA Vaccines

Production of DNA vaccines starts with *E. coli *cells which are transformed with the plasmid of interest. These cells are grown and stored frozen in a stock of vials called a Master Cell Bank. Growth of the *E. coli *is typically done via a fermentation process similar to that used in the manufacturing of certain alcoholic beverages. The recovery process then requires lysis of the cells, in order to release the plasmid retained within the *E. coli *cells. DNA is then purified using various chromatographic methods.

### Advantages

DNA vaccination has many advantages compared with conventional vaccine approaches (Appendix 1) particularly in the setting of protecting against potentially lethal emerging infectious diseases. Protecting against a particular pathogen may require immunity to more than one component of the organism and may require stimulation of different components of the host immune system. Traditionally, most preventive vaccines have relied on antibodies as the main correlate of protection, components that prevent infection or disease. However, T cells play an important role in controlling disease for established infection. Conventional vaccines based on whole pathogens typically induce immune responses against a number of irrelevant components of the organism. Subunit protein vaccines target individual components of the pathogen and usually only stimulate antibodies. DNA vaccines can accommodate a combination of different genes that code for different antigens from one or more different pathogens. This can result in the generation of broad immunity to multiple protein antigens. DNA vaccines have also been observed to stimulate both antibody and T cell arms of the immune system including those that are specialized to kill viruses or cancer cells (via cytotoxic or killer T cells). A significant advantage, especially for emerging pathogens, is that DNA vaccines do not require the handling of potentially deadly infectious agents. In addition they have a significantly shorter production time, something paramounrt with an ongoing pandemic.

### Safety

Overall, a well-tolerated safety profile has been observed in human clinical trials of DNA vaccination [10]. Early in the development, there were numerous theoretical safety concerns regarding DNA vaccines. In particular, there were concerns about the fate of the injected genes and the potential for insertion into the host DNA possibly resulting in the uncontrolled stimulation of other genes such as those that may cause cancer. These concerns have dissipated over the years based on thousands of human subjects who have undergone DNA vaccination or plasmid-based gene therapy. Furthermore, in pre-clinical safety studies in animals, the potential for plasmid integration into the host genome has been shown to be negligible and several orders of magnitude below the spontaneous mutation rate that occurs naturally in mammalian genes [11]. There were also early concerns regarding the induction of autoimmune reactions, as DNA may be considered as a self antigen to the host. Increased rates of autoimmunity or anti-DNA antibodies such as those observed in Systemic Lupus Erythematosis have not been observed in clinical trials of DNA vaccination [12]. Interestingly, as plasmid DNA vaccines are noninfectious, significant immune responses are not developed against the plasmid itself. Therefore only specific immune responses to the expressed antigen are stimulated with this approach. In contrast, with viral vectors such as adenoviruses, pre-existing and post vaccination immunity to the vector itself can be generated thereby limiting the pathogen specific immune response In contrast, DNA vaccination can be repeated without diminishing the specific immune response. This may be a pertinent beneficial aspect of DNA immunization, particularly in light of recent clinical trials in humans using viral vector-based vaccines, such as the common cold adenovirus, where immunity to the vector itself has been raised as a potential safety issue [13,14]. In summary, the potential risks of DNA vaccines appear to be minimal based on safety data from human clinical trials in thousands of subjects.

### Recent Advances in Optimizing Immune Responses to DNA Vaccines

Over the years, much progress has been made in optimizing DNA vaccine immunogenicity. Recent progress has targeted many different aspects of DNA vaccination which has successfully resulted in improved immune responses (Appendix 2).

### Plasmid Optimization

With regard to the plasmid itself, significant advances have been made in optimizing the genetic sequence of the encoding gene as well as other related components. Co-administration of the plasmid that encodes the pathogen along with other genes that encode for immune stimulating substances such as cytokines or chemokines has also been used to further enhance the immune response of certain DNA vaccines [15].

In some cases, once various components of the plasmid have been optimized, the encoded protein may be sufficiently immunogenic without the need for additional components (unformulated or "naked" DNA) such as adjuvants. One of the earliest trials in humans of an unformulated or "naked" DNA vaccine was one that targeted the malaria parasite. Wang and colleagues at the Naval Medical Research Institute immunized 20 subjects with a plasmid that encoded naturally occurring forms of malaria proteins [16]. In this trial, more than half of the subjects were shown to have cells that can kill or lyse malaria infected cells (cytotoxic T cell or killer cells). In a more recent clinical trial, a DNA vaccine optimized for human expression and encoding modified forms of West Nile Virus proteins was studied by Martin and colleagues at the National Institute of Health (NIH) and demonstrated that the vaccine stimulated antibodies that inhibited the virus (neutralizing antibodies) in all individuals receiving the vaccination regimen [17]. This unformulated DNA vaccine appears to induce a similar level of immune responses to those observed in vaccinated horses protected from WNV. In addition, a recent clinical trial by these same NIH researchers tested an optimized but unformulated DNA vaccine for SARS coronavirus and demonstrated neutralizing antibodies in all subjects who received three doses of the vaccine [18].

### Route of Delivery

Another area of research to enhance the immune response to DNA vaccines is related to the route of administration. Although intramuscular injection is the predominant route of vaccine delivery, other routes of administration have also been studied such as intradermal delivery. It is conceivable that intradermal delivery, which is a more superficial injection, may result in better transfection of antigen presenting cells, particularly a type of cell called dendritic cells.

### Mode of Delivery

The mode of delivery may also be a pertinent factor in eliciting the proper immune response after DNA immunization.

Needle and syringe is the predominant method to deliver both DNA vaccines as well as conventional vaccines. However, Roy and colleagues at PowderMed (now Pfizer Incorporated) have used DNA precipitated onto gold particles which are driven into to skin with a blast of pressurized gas, and called this approach "particle-mediated epidermal delivery" (PMED). In one clinical study by Roy and collaborators using PMED, individuals exhibited potent antibody responses to a hepatitis B DNA vaccine [19]. In another study, Drape and colleagues at PowderMed also observed strong antibody responses to the influenza virus DNA vaccine with the PMED approach [20].

Another approach for the delivery of DNA vaccines is the use of needle-free devices. Needle-free injection of DNA vaccines has been utilized in numerous clinical studies and appears to be well-tolerated and may have some advantages of further augmenting the immune response to DNA vaccination. For example, Nabel and colleagues at the NIH injected individuals intramuscularly with a six-plasmid unformulated DNA vaccine for HIV (Env A, Env B, Env C, subtype B gag, Pol, and Nef) with the needle-free device (Biojector^® ^2000) and observed HIV specific T-cell immune responses in over 75% of individuals[21]. Similarly, this same group at the NIH completed a recent study of an Ebola DNA vaccine also using this same needle-free device. They demonstrated that Ebola-specific antibody and CD4+ T-cell immune responses were elicited in all individuals who received the three-dose vaccination regimen [22].

Another novel mode of enhancing DNA vaccines has been a more invasive technique called electroporation. This method involves administration of brief electrical pulses of various voltages, after injection of a DNA vaccine, in order to enhance the uptake of DNA, presumably through the formation of transient pores in the muscle cell membrane. Many groups have shown encouraging results using electroporation in animals. One of the first groups to use this technique, Ulmer and colleagues, at Chiron Corporation, demonstrated that antibody and T-cell responses to the HIV protein Gag encoded in a DNA vaccine were enhanced by electroporation in rhesus macaques [23]. More recently, studies by Pavlakis and colleagues at the NIH have also noted that DNA potency measured by T-cell responses to the HIV protein Gag was augmented in nonhuman primates using electroporation [24]. Although encouraging in the strong magnitude of responses generated in animals, the wide clinical applicability of electroporation in humans in relation to tolerability remains to be determined.

Some researchers have used plasmid DNA in what has been called a heterologous "prime-boost" vaccination approach. This method involves delivery of one or more plasmid DNA vaccine priming doses followed by a boost with a viral vector (such as adenovirus) which codes for the same antigens. In the prime-boost setting, DNA vaccination plays an important role in priming different types of T-cells (CD4+ and CD8+ T-cells) specific for various proteins. In studies, for example, by Nabel and colleagues at the NIH, DNA vaccination with plasmids encoding HIV antigens followed by a boost with an adenoviral vector carrying the same HIV gene sequences, resulted in stronger T-cell responses compared with adenoviral vector or DNA vaccine alone [25]. Similarly, Pantaleo and colleagues at the University of Lausanne have shown that a DNA vaccine for HIV followed by a boost with a poxvirus vector (NYVAC) resulted in stronger immune responses compared to immunization with pox vector alone [26]. In another prime-boost study, Jacobson and colleagues, at the University of California at San Francisco, immunized subjects with a DNA vaccine for CMV who were then boosted with a live-attenuated CMV virus (Towne strain). Faster and stronger virus-specific T-cell responses were observed in the group of subjects that received the DNA and Towne strain compared with a group of subjects that received the Towne strain alone [27].

### Formulated DNA Vaccines

An area of potentially paramount importance for DNA vaccines is formulations and adjuvants. Adjuvants are common to most licensed vaccines and are included to potentiate the immune responses elicited by vaccination. For DNA vaccines, various delivery systems and adjuvants have been tested. One of the earliest promising adjuvants for plasmid DNA vaccines was poly-lactide coglycolide (PLG), cationic microparticles. Ulmer and colleagues at Chiron Corporation evaluated HIV DNA vaccines formulated with PLG microparticles and found strong antibody and T-cell responses in macaques [28]. Poloxamers represent another class of adjuvants tested with DNA vaccines. Some poloxamers are nonionic block copolymers, and when combined with a cationic surfactant, bond with DNA to form small particles. A study by Wloch and colleagues at Vical Incorporated examined DNA immunizations of human volunteers with cytomegalovirus (CMV) plasmids formulated with a specific poloxamer adjuvant. In that study CMV-specific T-cell responses were detected in a majority of CMV sero-negative individuals who were vaccinated [29].

Vaxfectin^® ^is another example of a delivery system and adjuvant for DNA vaccines that has been recently tested in humans. Vaxfectin^® ^is a cationic lipid- based adjuvant that bears a positive charge that binds electrostatically to negatively charged DNA. Studies in animals also demonstrated that Vaxfectin^®^-adjuvanted DNA vaccines can be protective against lethal viral challenges. For example, Webby and colleagues at St. Jude Children's Research Hospital and Vical Incorporated, immunized ferrets with three plasmids containing DNA components of the H5N1 pandemic influenza virus formulated with Vaxfectin^® ^[30]. After one or two immunizations, all animals were completely protected from lethal pandemic influenza virus challenge, while unvaccinated control animals died. Similarly, Griffin and colleagues at Johns Hopkins immunized juvenile and infant rhesus macaques by intramuscular and intradermal routes with measles antigen encoding plasmids formulated with Vaxfectin^® ^[31]. All of the vaccinated monkeys developed strong and durable neutralizing antibodies and they were challenged with high doses of measles virus after one year. All of the unvaccinated control animals developed viremia and became ill with rashes in contrast to the vaccinated animals which remained healthy and had no detectable virus levels. Lastly, the first human clinical trial of a DNA vaccine formulated with Vaxfectin^® ^has been completed with plasmids that encode pandemic influenza virus antigens (H5N1). The preliminary results reported from this trial by Smith and colleagues at Vical Incorporated suggest that vaccination with H5 DNA plasmids formulated with this adjuvant were well-tolerated and stimulated strong H5 antibody responses in up to 67% of subjects [32]. Notably, the antibody response rate and safety profile observed in this trial are comparable to most conventional protein-based vaccines for pandemic influenza.

### Approved DNA Vaccines

Although DNA vaccines have yet to be approved for human use, three have already been approved for animal health.

As noted in Appendix 3, the first DNA vaccine approved for animal health was one that protected horses against WNV. WNV is a mosquito-borne virus, which causes encephalitis or inflammation in the brain of infected animals and humans. Prior to approval of a vaccine, approximately one-third of the horses infected with WNV would die or be euthanized. The WNV DNA vaccine, developed by Fort Dodge Laboratories, was approved by the U.S Department of Agriculture (USDA) in 2005 after the demonstration of safety and efficacy[33].

A DNA vaccine has also been recently approved to prevent a fatal viral disease that afflicts salmon, called infectious hematopoietic necrosis virus. In mid-2001, an epidemic occurred in Atlantic salmon killing up to 90% of the fish. Scientists at Aqua Health, a unit of Novartis in Canada, conducted a field trial by immunizing millions of salmons with a single dose of DNA vaccine encoding for a protein of the virus [34]. The vaccine was approved in 2005 based on the results of this trial that demonstrated that the vaccine, called Apex-IHN, protected the fish from death without adverse effects.

Additionally, a therapeutic DNA vaccine designed to treat dogs with skin cancer (melanoma) was granted conditional approval in 2007. This vaccine was developed through a collaboration of Memorial Sloan-Kettering Cancer Center (MSKCC) and Merial Ltd. Canine melanoma is an aggressive form of cancer. Dogs with melanomas that have gone beyond initial stages typically have a lifespan of one to five months with conventional therapies. In addition, the cancer is often resistant to chemotherapy. In a study of the DNA vaccine conducted by MSKCC, many dogs who received the vaccinations lived beyond the average 13 month survival [35]. Based on the significantly extended survival, the USDA gave this DNA vaccine conditional approval in 2007. This is the first therapeutic vaccine approved by the U.S. government for the treatment of cancer in animals or humans.

### Vaccine Production Time for Emerging Infectious Diseases

Perhaps most relevant to emerging infectious diseases, DNA vaccines have the distinct advantage of a rapid development time, are non- infectious, and have a well-defined manufacturing process. DNA vaccines contain no infectious components and can be produced safely without the handling of hazardous infectious agents. Furthermore, there is a well-defined analytical process for the manufacturing of all DNA vaccines, which is universally applicable to any DNA vaccine.

**Figure 3:**
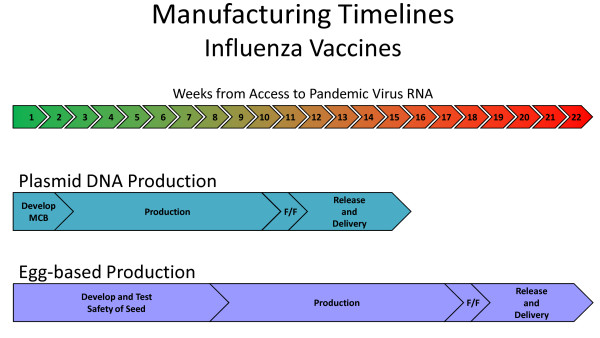
**Manufacturing timelines for DNA vaccines compared to egg-based protein vaccines**. F/F = fill finish.

Vaccination is an important component of a response to potential pandemics such as avian influenza. According to the World Health Organization (WHO), since November 2003, approximately 400 cases of human infection with highly pathogenic avian influenza A (H5N1) have been reported worldwide [36]. Pandemic (H5N1) influenza virus has evolved into at least 10 distinct clades or subclades. As noted earlier, the emergence of triple-reassortment swine influenza with limited cross reactivity antibody responses after vaccination with seasonal influenza vaccines suggests the need to rapidly produce new vaccines to this particular emerging virus [37]. The manufacturing time of conventional protein-based vaccines may be excessive, as they typically require growth in egg or cell cultures, which involve a relatively slow production time. DNA vaccines, in contrast, have estimated vaccine production times that can be months earlier, as only the DNA sequence is required and the manufacturing process is standard (Figure 3) [38]. DNA vaccines therefore have a unique advantage of large scale production for human use in a relatively streamlined period of time. In the case of potentially fatal emerging pathogens, reducing the production time of an effective vaccine may be critical in preventing spread of infection and death.

In summary, based on recent advances in enhancing protective immune responses and a well-tolerated safety profile in humans, plasmid DNA vaccines have the potential to become an integral part of the arsenal dedicated to enhancing human health by preventing diseases through immunization in our ever changing microbial environment.

## Competing interests

The author declares that they have no competing interests.

## Appendix 1: Potential advantages of DNA vaccines for emerging pathogens

Ability to immunize against multiple antigens and/or pathogens

Ability to stimulate both T-cell and antibody immunity

Safety profile

Nonviral and no induction of anti-vector immunity

Ability to repeat injections (IM or ID)

Strong priming effect

Large rapid GMP manufacturing capabilities

No need to handle infectious pathogens during production process

## Appendix 2: Optimizing immune responses elicited by DNA vaccination

• DNA sequence or promoter

• Route

◦ IM

◦ ID

• Mode of delivery

◦ Needle/syringe

◦ Particle-mediated epidermal delivery

◦ Needle-free

◦ Electroporation

• Adjuvant

◦ Cationic microparticles (e.g., PLGA)

◦ Nonionic block copolymers (e.g., poloxamer)

◦ Cationic lipid systems (e.g., Vaxfectin^®^)

• Prime-boost

## Appendix 3: Approved or conditionally DNA vaccines for animal health

### Infectious Disease Vaccines

• Infectious hematopoietic necrosis virus in salmon (Novartis Animal Health)

• West Nile virus for horses (Fort Dodge Animal Health)

### Cancer Vaccine

• Melanoma for dogs - conditional approval (Merial LTD)
